# A review of the genus *Raveniola* (Araneae, Nemesiidae) in China, with notes on allied genera and description of four new species from Yunnan

**DOI:** 10.3897/zookeys.211.3060

**Published:** 2012-07-25

**Authors:** Sergei Zonstein, Yuri M. Marusik

**Affiliations:** 1 Department of Zoology, The George S. Wise Faculty of Life Sciences, Tel-Aviv University, 69978 Tel-Aviv, Israel; 2 Institute for Biological Problems of the North RAS, Portovaya Str. 18, Magadan, Russia; 3 Zoological Museum, University of Turku, FI-20014 Turku, Finland

**Keywords:** Araneae, spiders, taxonomy, Nemesiidae, *Raveniola*, *Sinopesa*

## Abstract

The Chinese representatives of *Raveniola* Zonstein, 1987 are currently recognized to comprise seven species. Four new species – *Raveniola montana* **sp. n.** (♂♀), *Raveniola shangrila* **sp. n.** (♂), *Raveniola songi* **sp. n.** (♂) and *Raveniola yunnanensis* **sp. n.** (♂) – are described from the highlands of Yunnan Province, China. According to some characters (shape of the palpus, palpal tibia and tibia I in males) they can be placed together with *Raveniola hebeinica* Zhu, Zhang & Zhang, 1999 and with *Raveniola guangxi* (Raven & Schwendinger, 1995), **comb. n.**, transferred here from *Sinopesa* Raven & Schwendinger, 1995. The current generic position of *Raveniola xizangensis* (Hu & Li, 1987) is confirmed. Other Chinese nemesiids referred previously to *Raveniola* are transferred to *Sinopesa*: *Sinopesa chinensis* (Kulczyński, 1901), **comb. n.**, *Sinopesa sinensis* (Zhu & Mao, 1983), **comb. n.** and *Sinopesa chengbuensis* (Xu & Yun, 2002), **comb. n.** The relationships between these Asian genera and their relations to Afrotropical nemesiids are discussed.

## Introduction

*Raveniola* Zonstein, 1987 with 20 named species is the fifth largest genus of the globally distributed Nemesiidae, encompassing 356 species belonging to 43 genera (Platnick 2012). The genus is restricted to the south Palearctic, chiefly to mountainous regions, and occurs from Turkey to south China. Most species are local endemics and fairly evenly distributed through the range. *Raveniola* has never been subject to revision. Only two species, *Raveniola concolor* Zonstein, 2000 and *Raveniola vonwicki* Zonstein, 2000, were described in the same paper. All other species were described in separate papers using different styles and involving different sets of characters, hindering, if not preventing, identification and proper comparison of species.

Among the countries the highest number of *Raveniola* species, five out of 20, is reported from China (cf. Platnick 2012). However, this figure can not be considered as high when considering the size of the country and habitat diversity. The much smaller Georgia is inhabited by three species (cf. Mikhailov 1997), while Central Asia, which is comparable in size to China, harbors nine named *Raveniola* species (cf. Mikhailov 1997; Platnick 2012) and also several undescribed ones (Zonstein, personal data). A recent opportunity to study a little material from south China revealed four undescribed species. To enable description we initiated a review of all nemesiids described or reported from China and compared *Raveniola* with another genus, *Sinopesa* Raven & Schwendinger, 1995, occurring in China. The main aims of this paper are thus as follows: to provide 1) a key to all *Raveniola* species known from China, 2) their diagnoses as well as descriptions of new species, 3) a delimitation of two related genera *Raveniola* and *Sinopesa*, and 4) the correct allocation of Chinese nemesiids belonging to these genera.

## Material and methods

The study began with an examination of several nemesiid series donated to us by Russian entomologists who had visited Yunnan Province in the People’s Republic of China in 2005. One of us (YM), had additionally collected nemesiid material while visited China in 2011. One species (*Raveniola hebeinica*) was obtained courtesy of our Chinese colleagues (Shuqiang Li and Zhang Feng). A rich collection of comparative material, including the majority of known *Raveniola* species, as well as representatives of the nemesiid genera *Hermacha* Simon, 1889, *Entypesa* Simon, 1902, *Lepthercus* Purcell, 1902, *Pionothele* Purcell, 1902and *Sinopesa* (4, 8, 1, 1 and 3 species, respectively), was obtained from the collections listed below.

Institutional acronyms used here are: **ARC** – Agriculture Research Council, Pretoria, South Africa; **BDSU** – Biology Department of Shandong University, China; **FMNH** – The Field Museum of Natural History, Chicago, USA; **HUB** – Hebei University, Baoding, China; **IZAS** – Institute of Zoology, Chinese Academy of Sciences, Beijing, China; **MHNG** – Muséum d’histoire naturelle, Genève, Switzerland; **MNHN** – Muséum national d’Histoire naturelle, Paris, France; **NHM** – Natural History Museum, London, UK; **NMW** – Naturchistorisches Museum Wien, Austria; **TAU** – Zoological Museum, Tel Aviv University, Israel; **ZMMU** – Zoological Museum of Moscow University, Russia.

Other abbreviations are as follows. *Eyes*: **ALE** – anterior lateral; **AME** – anterior median, **PLE** – posterior lateral, **PME** – posterior median. *Spinnerets*: **PLS** – posterior lateral, **PMS** – posterior median. *Spine shape and position*: **d** – dorsal; **M** – megaspine**; p** – prolateral; **pd** – prodorsal; **pv** – proventral; **r** – retrolateral; **rd** – retrodorsal; **rv** – retroventral; **v** – ventral.

**Figure 1.**
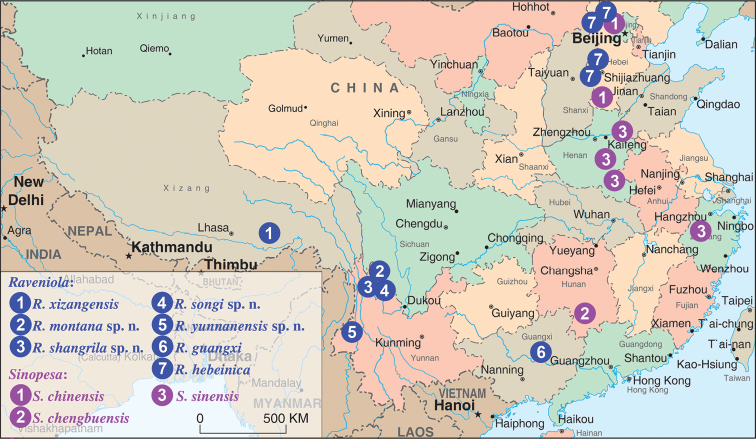
Localities of *Raveniola* and *Sinopesa* species in China. ***Raveniola*:** **1** XIZANG/TIBET: Jansha County **2–4** YUNNAN: Shika Mts. **5** YUNNAN:Finchuiyanou Mts. **6** GUANGXI: Liuzhou **7** BEIJING municipality: Mt. Xialongmen; HEBEI: Tuoliang, Bai’an, Damaqun Shan Mts. ***Sinopesa*:** **1** BEIJING municipality: Chanping; HEBEI: Pingxiang **2** HUNAN: Chengbu County **3** ZHEJIANG: Lin’an; HENAN: Huaiyang, Xiping, Yueyang, Shāngchéng.

Photographs were taken using a Canon 500D digital camera with a 100 mm Canon macro lens and a Zeiss Discovery V20 stereomicroscope with a Canon PowerShot G9 digital camera attached to it. Measurements were taken to an accuracy of 0.01 mm. All measurements are given in millimetres. Total body length includes chelicerae but not spinnerets. Diameter of AME is given usually as a diameter of a sharply edged AME pupil. When the eye dome was mounted well and could be measured, the corresponding data follow in parentheses. Any measurements for this parameter are also given in parentheses. The length of sternum was measured along the straight line between the posterior tip of the sternum and the hindmost part of the labium. Lengths of leg and palp segments were measured on the dorsal side, and lengths of spinneret segments on the ventral side, from midpoint of anterior margin to midpoint of posterior margin. Lengths of palps and legs are given as: total (femur, patella, tibia, metatarsus and tarsus). Fig. 1 was created on the base of a small tourist map located online at http://www.homepages.ucl.ac.uk/~zczcc07/maps.htm and claimed to be free to reproduce and use.

## Taxonomy

### Raveniola

Zonstein, 1987

http://species-id.net/wiki/Raveniola

Raveniola Zonstein 1987: 1014, type species *Brachythele virgata* Simon, 1891, by the original designation.

#### Diagnosis.

By retroventral position of the male mating spur on tibia I, *Raveniola* differs essentially from the majority of the Holarctic and Asian nemesiid genera: from Mediterranean *Nemesia* Audouin, 1826, *Iberesia* Decae & Cardoso, 2006 and *Brachythele* Ausserer, 1871 as well as from the Nearctic *Calisoga* Chamberlin, 1937 and from Asian *Atmethochilus* Simon, 1887 and *Damarchus* Thorell, 1891. Males in all these genera possess mating spurs located on the process ventrally or prolaterally. In addition, in the two latter genera males have paired tarsal claws provided with a single S-shaped tooth row instead of the biserial dentition typical for male nemesiids.

Within the rest of this group of genera, in which males are also known to possess the retrolateral or retroventral megaspines on tibia I, *Raveniola* can be distinguished from Central American *Mexentypesa* Raven, 1987 by having the unpaired tarsal claw (absent in the latter genus) and integral tarsi (pseudosegmented in *Mexentypesa*); from African *Hermacha* Simon, 1889, *Entypesa* Simon, 1902, *Lepthercus* Purcell, 1902 and *Pionothele* Purcell, 1902 – by much smaller PMS (from first three of them) or longer apical segment of PLS (domed in *Pionothele*). Moreover, males of *Raveniola* differ from males of all the above-mentioned genera by their elongate, cylindrical and strongly spinose palpal tibiae. The congeneric females have no unique distinctive characters.

East-Asian *Sinopesa* Raven & Schwendinger, 1995 is the only genus that has been found to share with *Raveniola* the above-listed definitive features. These partially sympatric genera differ from each other by the characters shown in the table in the Discussion below.

#### Description.

Medium-sized to large nemesiids with carapace 4–14 mm long. Carapace hirsute. Eye tubercle low to moderately developed. Chelicerae in most species without rastellum. Maxillae rectangular with few to numerous cuspules. Serrula not evident. Labium twice wider than long with no cuspules. Paired sternal sigillae small round submarginal to marginal. Leg formula 4123 or 1423. Metatarsal preening combs absent. Leg tarsi integral (not cracked or pseudosegmented), aspinose in most species. In males scopula on tarsi I always entire; tarsi II with entire or narrowly divided scopula; tarsi III and IV with widely divided scopula or without it; conspecific females with weaker scopula on posterior tarsi. Paired tarsal claws biserially toothed both in males and females; claw apex long and moderately curved. Unpaired tarsal claw small and sharply bent. Males: intercheliceral tumescence if present located ventrally; palpal tibia ±long, spinose; cymbium rather short with or without spines; tibia I with 2(3) sequential megaspines. Females: each paired spermatheca with 2–3 individual diverticulae. PMS small to absent. PLS: apical segment triangle to digitiform.

#### Distribution, habitats and ecology.

Over 20 species are currently known in the south Palearctic, from Turkey to China (see Platnick 2012). The spiders inhabit different types of forest and steppe biotopes, subalpine and alpine meadows from seashore up to 4300 m above sea level. They can occur under rocks and logs, or inhabit abandoned rodent burrows, or crevices. Adult females can be found building simple burrows 10–20 cm (in *Raveniola ferghanensis* - up to 40–50 cm) depth with weak silk lining and open entrance.

Unfortunately, we have no direct label data shoving peculiarities in the habitats and ecology of Chinese species of *Raveniola*. The only male of *Raveniola yunnanensis* sp. n.was found under the rock in the mixed mountainous broad-leaved forest (I. Kabak, personal communication). The representatives of *Raveniola montana* sp. n., *Raveniola shangrila* sp. n. and *Raveniola songi* sp. n. were collected with pitfall traps together with highland ants *Myrmica pleiorhytida* Radchenko & Elmes, 2009 (Hymenoptera, Formicidae) by the same collectors and in the same biotope. The latter species was noted inhabiting mountainous meadows (see Radchenko and Elmes 2010, p. 219).

#### Key to Chinese *Raveniola*

Females of *Raveniola guangxi*, *Raveniola shangrila* sp. n., *Raveniola songi* sp. n. and *Raveniola yunnanensis* sp. n. are unknown.

**Table:** 

1	Large species: carapace length > 10 mm. Males: embolus with subapical keel (Fig. 49). Females: basal receptacle bifurcate (Fig. 50)	*Raveniola xizangensis*
–	Smaller: carapace length 4.6–7.3 mm. Males: subapical embolic keel absent or vestigial. Females: basal receptacle entire	2
2	Males	3
–	Females	7
3	PMS absent	4
–	PLS (sometimes tiny) present	5
4	Maxillae with few (3–4 in the holotype) cuspules. Embolus as shown on Figs 37 and 38	*Raveniola guangxi*
–	Maxillae with numerous (>13) cuspules. Embolus as in Fig. 41	*Raveniola shangrila* sp. n.
4	PLS: apical segment digitiform. Embolus ±twisted (Figs 40–43)	5
–	PLS: apical segment triangle. Embolus curved apically (Fig. 39)	*Raveniola hebeinica*
5	Leg I: tibia incrassate, equal in length with, or shorter than metatarsus (Figs 26, 28). Few spines on cymbium (Figs 32–34)	6
–	Leg I: tibia very long and slender, considerably longer than metatarsus (Fig. 29). Cymbium with numerous dorsal spines (fig. 35)	*Raveniola yunnanensis* sp. n.
6	Leg I: metatarsus straight, longer than tibia (Fig. 26). Cymbium with long spines (Fig. 32). Embolus short with well developed embolic ridges (Fig. 40)	*Raveniola montana* sp. n.
–	Leg I: metatarsus ±curved, equal in length to tibia (Fig. 28). Spines on cymbium shorter (Fig. 34). Embolus longer without or with very weak embolic ridges (Fig. 42)	*Raveniola songi* sp. n.
7	PLS: apical segment triangle. Spermathecae as shown in Fig. 47	*Raveniola hebeinica*
–	PLS: apical segment digitiform. Spermathecae as shown in Fig. 48	*Raveniola montana* sp. n.

### Raveniola guangxi

(Raven & Schwendinger, 1995) comb. n.

http://species-id.net/wiki/Raveniola_guangxi

Figs 7 15 24 30 37, 38

Sinopesa guangxi Raven & Schwendinger, 1995: 633–635, figs 3C, 4B, 4E, 8A–E (♂); Song at al. 1999: 40, fig. 17K (♂).

#### Types.

Holotype ♂ – CHINA: GuangxiProvince: Liuzhou, Dragon Lake (24°16'N, 109°24'E); in MCZ; examined.

#### Diagnosis.

Differs from all other known Chinese congeners except *Raveniola shangrila* sp. n. by absence of PMS. From the latter species *Raveniola guangxi* may be distinguished by shape of the embolus and by fewer maxillary cuspules – 3–4 vs. 15–20 (cf. Figs 15, 30, 37 and 19, 33, 41, respectively).

#### Description.

The holotype male was described in detail by Raven & Schwendinger, 1995. Carapace, sternum with labium and maxillae, tibia and metatarsus I, palpal tibia and cymbium, and bulbus (in retrolateral and ventral aspects) as shown in Figs 7, 15, 24, 30, 37 and 38. Female unknown.

#### Distribution.

Known only from the type locality (Fig. 1).

### Raveniola hebeinica

Zhu, Zhang & Zhang, 1999

http://species-id.net/wiki/Raveniola_hebeinica

Figs 8 16 25 31 39 47

Raveniola hebeinica Zhu et al. 1999: 366, figs 1–10 (♂♀); Song et al. 2001: 56, figs 22A–I.

#### Types.

Holotype ♂ and paratypes ♀♀ from Mt. Tuolang (Hebei Province, Pinshang County, 38°45'N, 113°49'E, 1500–2000 m); supposed to be in HUB, but was not found (Feng Zhang, personal communication).

#### Material examined.

Beijing Municipality, Mt. Xialongmen (39°58'N, 115°27'E), 1000–1300 m, 60–65 km W Beijing, 21–23.09.2001, coll. Y. D. Yu – 1♂ (IZAS). Hebei Province: Xingtai County, Taihang Mts., surroundings of Bai’an 60 km W Xingtai City (approximately 37°04'N, 113°48'E), 600–1000 m, 16.07.2007, coll. Jiao Guobin – 1♀, 1♀ subad. (HUB); Damaqun Shan Mts. (40°31'N, 115°49'E, 1000–1200 m) 75 km NW Beijing, 12.08.2010, coll. Yu. M. Marusik – 2♀ subad., 1 juv. (TAU).

#### Diagnosis.

The species differs from all known Chinese *Raveniola* species by small lateral receptacles in females (cf. Zhu et al. 1999, fig. 7) and by the subapically curved embolus in males (cf. Zhu et al. 1999, figs 8–10).

#### Description.

♂♀ were well described by Zhu et al. (1999).Male carapace, sternum with labium and maxillae, tibia and metatarsus I, palpal tibia and cymbium, bulbus and female spermathecae as shown in Figs 8, 16, 25, 31, 39 and 47, respectively**.**

#### Variability.

The only examined male has carapace 7.02 mm long (7.29 in the holotype). The carapace of the noticeably smaller examined adult female measures only 5.17 mm (vs. 6.67 in the female paratype used at the description).

#### Distribution.

CHINA: Hebei Province and Beijing Municipality (Fig. 1).

### Raveniola montana sp. n.

urn:lsid:zoobank.org:act:3EC3FB22-1D28-43BC-9A80-99C452367601

http://species-id.net/wiki/Raveniola_montana

Figs 5 9 10 17 18 26 32 40 48

#### Types.

Holotype ♂ and paratype ♀ – CHINA: Yunnan Province, Sueshan Mt. Ridge, Shika Mts. 10–15 km W Zhongdian (approximately 27°48'N, 99°35'E), 3800–4300 m, 25.05–6.06.2005, coll. I. Shokhin & S. Murzin (IZAS).

#### Etymology.

The specific epithet *montana* is derived from the Latin *montanus* (pertaining to the mountains), referring to the mountain habitat of this species.

#### Diagnosis.

The species differs from all other Chinese species of *Raveniola* by having a short embolus provided with deep ridges in males (Fig. 40); the specific configuration of female spermathecae is shown in Fig. 48.

#### Description.

Male (holotype). Body length 15.50. Colour in alcohol: carapace, chelicerae, palps and first pair of legs dorsally intense reddish brown; eye tubercle with darker spots surrounding AMEs and lateral eyes; sternum, labium, maxillae and legs II–IV light reddish brown; dorsal abdomen uniformly light greyish brown, ventral abdominal surface and spinnerets pale greyish brown.

General appearance as in Fig. 5. Carapace (Fig. 9) 6.35 long, 5.51 wide; covered with semi-adpressed dark hairs. Eye diameters (AME, ALE, PLE, PME): 0.19(0.26), 0.37, 0.19, 0.11. Interdistances: AME–AME 0.13(0.07), ALE–AME 0.09(0.06), ALE–PLE 0.04, PLE–PME 0.03, PME–PME 0.48. Cheliceral furrow with 9 promarginal teeth and 5 mesobasal denticles each. Labium (Fig. 17) 0.59 long, 1.08 wide. Maxillae with 15 cuspules each. Sternum 2.66 long, 2.65 wide. Palp: 8.66 (3.39, 1.59, 2.51, –, 1.17). Leg I: 20.23 (5.18, 3.06, 4.50, 4.82, 2.67). Leg II: 18.52 (4.97, 2.61, 4.13, 4.29, 2.52). Leg III: 18.15 (4.63, 2.48, 3.66, 4.65, 2.73). Leg IV: 23.41 (5.70, 2.66, 5.04, 6.88, 3.13). Leg I: tibia slightly incrassate, metatarsus slightly curved retroventrally (Fig. 26).

Spination. Palp: femur d1–1–1–1, pd1, rd1; patella p1–1–1, r1; tibia d1–1–2, p2–2–1, r0–1–1, v3–1–3; cymbium d5. Leg I: femur d1–1–1–1, pd1–1–1; rd 1–1–1; patella p1–1; tibia p2–1–0, pv1–1–0–0, rv1–1–M–M; metatarsus v0–0–2. Leg II:hg femur d1–1–1–1; pd1–1–1; patella p1–1; tibia p2(1)–1(0)–1, v2–2–3; metatarsus p1(0)–1–0; v2–2–3. Leg III: femur d1–1–1–1, pd0–1–1, rd0–1–1; patella p1–1, r1; tibia d1–1, p1–1–1, r1–1–1, v2–2–2(3); metatarsus d1–1–2, p1–1–1, r1–1–1, v2(3)–2–3. Leg IV: femur d1–1–0–0, pd0–1–1, rd0–1–1; patella p1, r1; tibia d1–1–2, p1–1–1, r1–1–1, v2–2–2(3); metatarsus pd1–1–2, p1–1–1, r1–1–1, v2–1–2–1(0)–3. Tarsi I–IV aspinose.

Scopula: distally on metatarsus I, entire on tarsus I, divided by setae on tarsus II; elsewhere absent. Paired claws: inner row with 5–6, outer row with 6–7 teeth. Trichobothria: 2 rows of 9–11 per row on tibiae, 12–17 on metatarsi, 11–14 on tarsi, 8 on cymbium.

Palpal tibia shortened, cymbium with few long spines (Fig. 32). Bulb provided with well-developed ridges; embolus short slightly twisted (Fig. 40).

Spinnerets. PMS: length 0.38; diameter 0.12. PLS: maximum diameter 0.35; length of basal, medial and apical segments 0.61, 0.63, 0.71; total length 1.95; apical segment digitiform.

Female (paratype): Body length 12.90. Colour in alcohol as in male, but slightly paler.

Carapace (Fig. 10) 4.66 long, 3.75 wide. Eye diameters (AME, ALE, PLE, PME): 0.16(0.20), 0.22/0.23, 0.14, 0.10. Interdistances: AME–AME 0.10(0.06), ALE–AME 0.07(0.05), ALE–PLE 0.05, PLE–PME 0.03, PME–PME 0.43. Cheliceral furrow with 9 promarginal teeth and 5 mesobasal denticles. Labium (Fig. 18) 0.50 long, 1.03 wide. Maxillae with 15 cuspules each. Sternum 2.16 long, 1.99 wide. Palp: 7.45 (2.64, 1.44, 1.74, –, 1.63). I: 12.00 (3.55, 1.70, 2.72, 2.33, 1.70). II: 10.86 (3.10, 1.63, 2.29, 2.17, 1.67). III: 10.62 (2.75, 1.49, 2.05, 2.60, 1.73). IV: 14.09 (3.70, 1.57, 3.01, 3.76, 2.05).

Spination. All femora with a few stiff bristles (undeveloped spines) located medially and distally; palpal patella, patella I and tarsi I–IV aspinose. Palp: femur pd0–0–1; tibia p2–2, v1–1–3; tarsus v2. Leg I: femur pd0–0–1; tibia v1(2)–1(2)–2; metatarsus v2–2–2. Leg II: femur pd0–0–1; patella p1; tibia p1–1, v1–1–2; metatarsus p0–1–0, v2–2–2. Leg III: femur pd 0–1–1, rd 0–1–1; patella p1, r1; tibia d0–1–1, p1–1–1, r1–1–1, v2–2–3; metatarsus d0–1–1, p1–1–1, r1–1–1, v2–2–3. Leg IV: femur pd0–1–1, rd0–0–1; patella r1; tibia d0–1–0, p1–1(0)–1, r0–1–1, v2–2–3; metatarsus d0–1–0, p1–1–1–1, r1–1–1, v2–1–2–3.

Scopula: distal on metatarsus I, narrowly divided by setae on palpal tarsus and tarsus I, widely divided and vestigial on tarsus II, elsewhere absent. Paired claws: promargin and retromargin on tarsi I and II with 6–7 teeth, on tarsi III and IV with 4–6 teeth each, respectively; palpal claw with 4 teeth on promargin. Trichobothria: 2 rows of 8–10 per row on tibiae, 14–16 on metatarsi, 11–12 on tarsi, 8 on palpal tarsus.

Spermathecae as in Fig. 48.

Spinnerets. PMS: length 0.32; diameter 0.13. PLS: maximum diameter 0.37; length of basal, medial and apical segments 0.59, 0.37, 0.50; total length 1.46; apical segment digitiform.

#### Distribution.

CHINA: Yunnan Province (Fig. 1).

**Figures 2–5.**
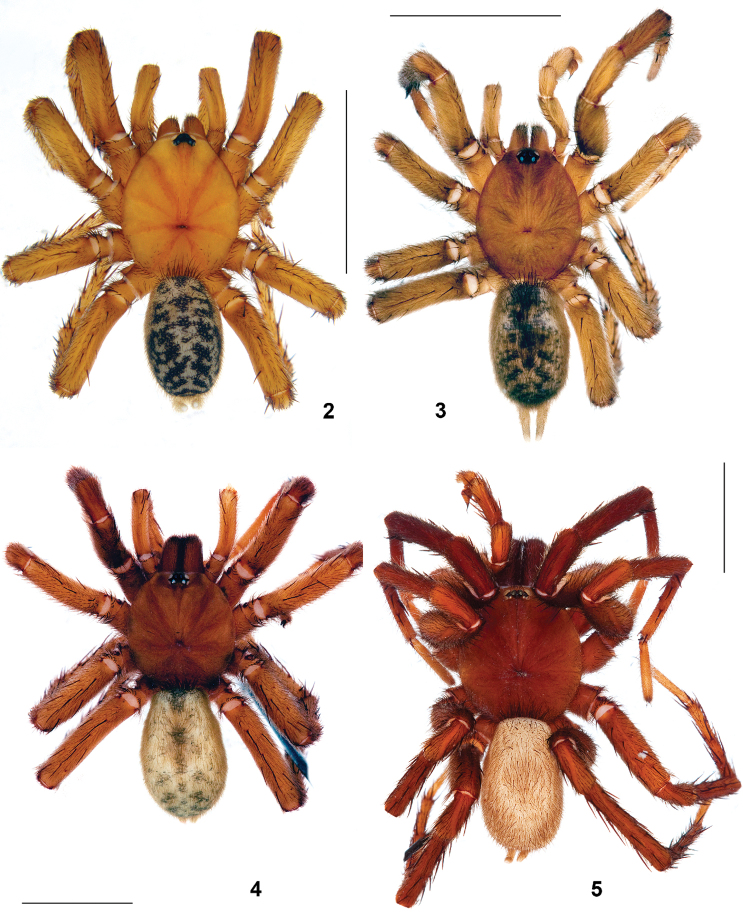
Male nemesiids, dorsal view. **2** *Sinopesa maculata*, Thailand **3** *Entypesa schoetedeni*, South Africa **4** *Raveniola virgata*, Kyrgyzstan **5** *Raveniola montana* sp. n., South China (scale bar = 5 mm).

**Figure:**
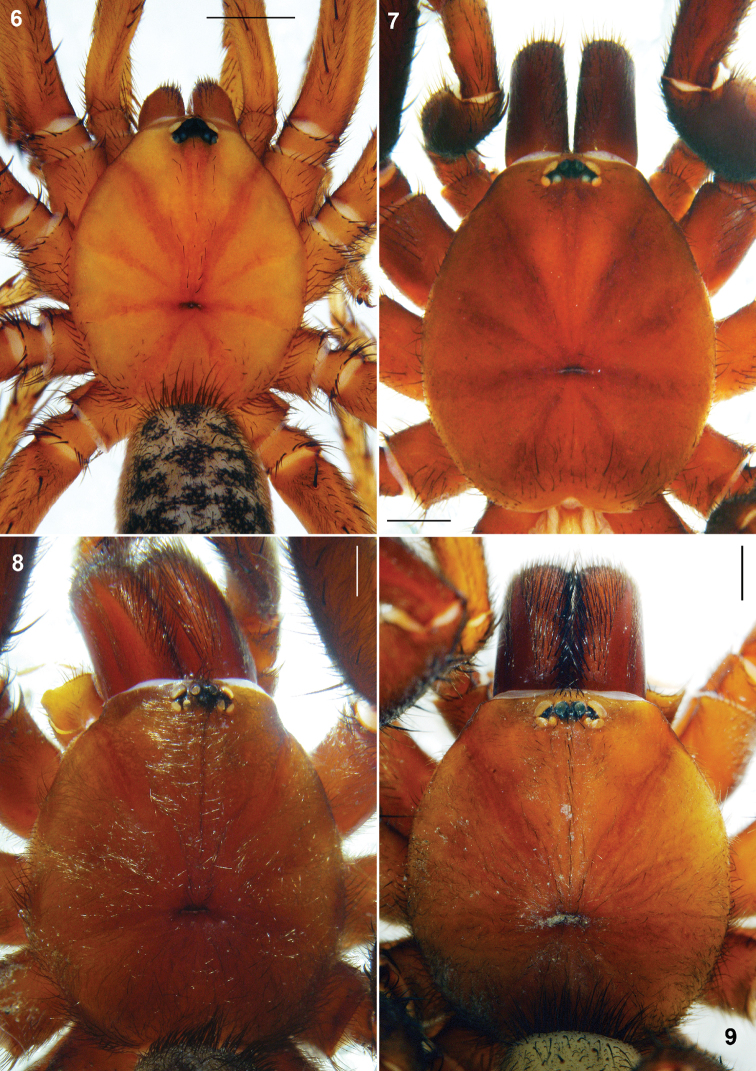
**Figures 6-9.** *Sinopesa* and *Raveniola*, holotype (**7, 9**) and conspecific (**6, 8**) males: carapace, dorsal view. **6** *Sinopesa maculata* **7** *Raveniola guangxi* **8** *Raveniola hebeinica* **9** *Raveniola montana* sp. n. (scale bar = 1 mm).

**Figures 10–13.**
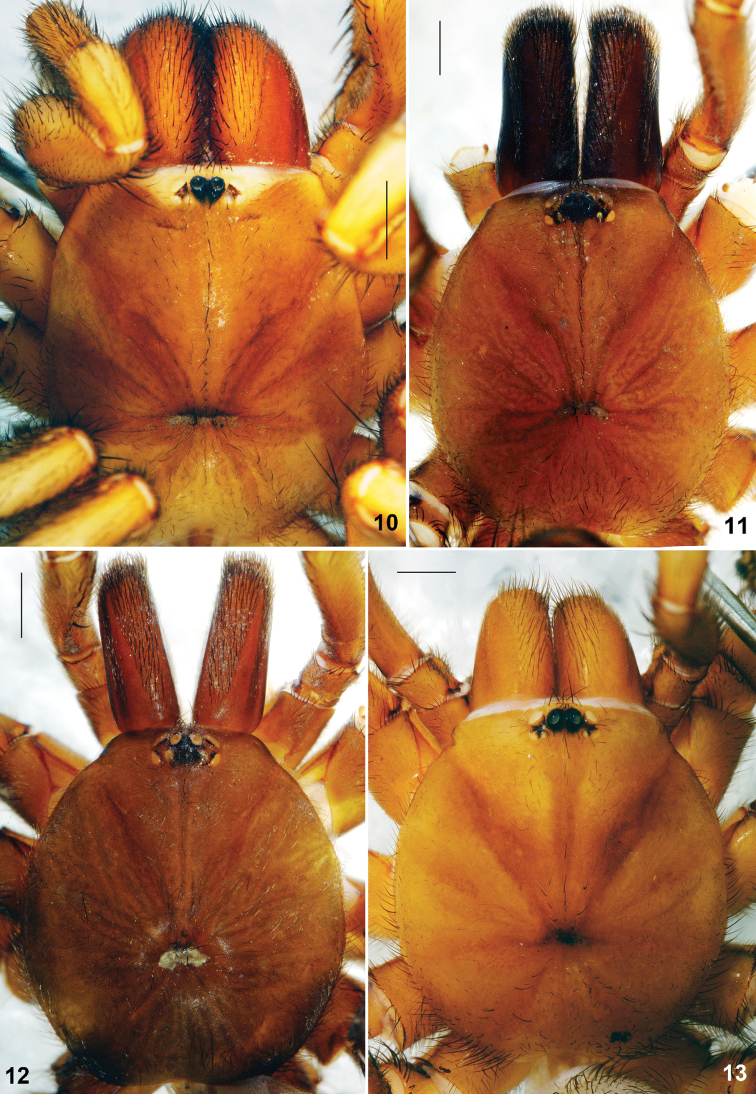
*Raveniola*, holotype (**13**) and paratype (**10–12**) males (**11–13**) and female (**10**) carapace, dorsal view **10** *Raveniola montana* sp. n. **11** *Raveniola shangrila* sp. n. **12** *Raveniola songi*sp. n. **13** *Raveniola yunnanensis* sp. n. (scale bar = 1 mm).

### Raveniola shangrila sp. n.

urn:lsid:zoobank.org:act:1C0D32BA-DC72-471C-847C-A783549E2A13

http://species-id.net/wiki/Raveniola_shangrila

Figs 11 19 27 33 41

#### Types.

Holotype ♂ – CHINA: Yunnan Province, Sueshan Mt. Ridge, Shika Mts. 10–15 km W Zhongdian (approximately 27°48'N, 99°35'E), 3800–4300 m, 25.05–6.06.2005, coll. I. Shokhin & S. Murzin (IZAS). Paratypes. 5♂ with the same collecting data are shared between MNHG, MNHN, NHML, TAU and ZMMU.

#### Etymology.

The specific epithet is given in honour of the mythical Tibetan land Shangri-La attributed to the highland region located in the far eastern part of Tibet (Xizang) and north-western part of Yunnan, i.e., including the type locality of this species.

#### Diagnosis.

The full reduction of PMS allows to place this species together with *Raveniola guangxi*; *Raveniola shangrila* can be distinguished from the latter species by shape of the embolus and larger number of maxillary cuspules – 15–20 vs. 3–4 (cf. Figs 15, 30, 37 and 19, 33, 41, respectively). In general, specimens of *Raveniola shangrila* sp. n. appear poorly distinguishable from those of *Raveniola songi* sp. n., but certain distinctions in the configuration of male bulb and metatarsus I are evident (cf. Figs 15, 16, 23 and 24).

#### Description.

Male (holotype). Body length 16.10. Colour in alcohol: carapace (with lighter spotted pattern), legs I partially, legs II–IV mostly middle foxy brown; sternum, labium and maxillae lighter coloured; chelicerae, all femora dorsally, tibiae and metatarsi I dark reddish brown; eye tubercle blackish brown; abdomen uniformly light brownish grey; genital area, booklungs and spinnerets pale yellowish grey.

Carapace (Fig. 11) 5.91 long, 5.45 wide; covered with semi-adpressed dark hairs. Eye diameters (AME, ALE, PLE, PME): 0.20(0.25), 0.31, 0.20, 0.20. Interdistances: AME–AME 0.15(0.11), ALE–AME 0.12(0.10), ALE–PLE 0.09, PLE–PME 0.03, PME–PME 0.57. Cheliceral furrow with 9 promarginal teeth and 4–5 mesobasal denticles. Labium (Fig. 19) 0.60 long, 1.03 wide. Maxillae with 17–19 small cuspules in wide triangle area. Sternum 2.85 long, 2.75 wide. Palp: 9.19 (3.56, 1.78, 2.77, –, 1.08). Leg I: 18.61 (5.02, 2.85, 4.09, 4.27, 2.38). Leg II: 16.11 (4.65, 2.56, 3.25, 3.46, 2.19). Leg III: 13.48 (3.71, 1.97, 2.47, 3.16, 2.17). Leg IV: 17.57 (4.72, 2.40, 3.81, 4.23, 2.41). Leg I: tibia incrassate, metatarsus curved retroventrally (Fig. 27).

Spination. All femora with a few stiff bristles (undeveloped spines) located medially and distally; patella IV and tarsi I–IV aspinose. Palp: femur pd1; patella p1; tibia d1–1–1, p1–1–1, r0–1–1, v1–2–0; cymbium d4. Leg I: femur pd1–0–1; patella p1; tibia p1–1–1, v2–2–M–M; metatarsus rv1. Leg II: femur pd1–0–1, rv0–0–1; tibia p1–1–1, v2–2–2; metatarsus p0–1–1; v2–2–3. Leg III: femur pd1–0–1, rd1–0–1; patella p1–1, r1–1; tibia d1–0, p1–1, r0–1, v2–2–2; metatarsus d0–1–1, p1–1–1, r0–1–1, v2–2–2(3). Leg IV: femur pd0–0–1, rd0–0–1; tibia p0(1)–1–1, r0–0(1)–1, v2–2–2; metatarsus p0–1–1, r0–1–1, v2–2–2.

Scopula: long, distal 1/2 on metatarsus I and II, entire on tarsi I and II, mixed with setae on tarsi III and IV. Paired claws with 6–8 teeth on promargin and retromargin. Trichobothria: 2 rows of 7–9 per row on tibiae, 12–15 on metatarsi, 10–12 on tarsi, 7 on cymbium.

Cymbium with few rather short spines (Fig. 33). Bulb without ridges; embolus long and twisted (Fig. 41).

Spinnerets. PMS: absent. PLS: maximum diameter 0.55; length of basal, medial and apical segments 0.84, 0.54, 0.75; total length 2.13; apical segment digitiform.

Female unknown.

#### Variability.

Carapace length in males varies from 5.03 to 5.95 (n=5).

#### Distribution.

CHINA: Yunnan Province (Fig. 1).

### Raveniola songi sp. n.

urn:lsid:zoobank.org:act:6B13CF73-BC7B-4901-85E6-5ED8DF4C8A9C

http://species-id.net/wiki/Raveniola_songi

Figs 12 20 28 34 42

#### Types.

Holotype ♂ – CHINA: Yunnan Province, Sueshan Mt. Ridge, Shika Mts. 10–15 km W Zhongdian (approximately 27°48'N, 99°35'E), 3800–4300 m, 25.05–6.06.2005, coll. I. Shokhin & S. Murzin (IZAS). Paratypes. 11♂ with the same collecting data are shared between IZAS (2), MNHG (2), MNHN (1), NHML (1), TAU (4) and ZMMU (1).

**Etymology.** The specific name is given in honour and memory of Prof. Daxiang Song (宋大祥; 1935–2008), for his immense contribution to Chinese arachnological research.

#### Diagnosis.

Males of *Raveniola songi* sp. n.habitually resemble those of *Raveniola shangrila* sp. n.but unlike them possess small PMS; other distinctive features are shown above (cf. Figs 15, 16, 23 and 24).

#### Description.

Male (holotype). Body length 13.50. Colour in alcohol: carapace, chelicerae and legs I medium reddish brown; eye tubercle somewhat darker; sternum, labium, maxillae and legs II–IV even lighter reddish brown; abdomen light greyish brown with darker pattern consisting of a weak longitudinal median spot and few transverse fasciae dorsally, and small irregularly shaped spots laterally and ventrally; genital area, book-lungs and spinnerets pale yellowish brown.

Carapace (Fig. 12) 5.10 long, 4.24 wide; covered with moderately dense and thin semi-adpressed dark hairs. Eye diameters (AME, ALE, PLE, PME): 0.18(0.24), 0.24, 0.20, 0.20. Interdistances: AME–AME 0.15(0.10), ALE–AME 0.06(0.04), ALE–PLE 0.08, PLE–PME 0.02, PME–PME 0.30. Cheliceral furrow with 9 promarginal teeth and 4–5 mesobasal denticles. Labium (Fig. 20) 0.33 long, 0.77 wide. Maxillae with 24–26 cuspules arranged in triangle area. Sternum 2.40 long, 2.28 wide. Palp: 7.86 (3.16, 1.59, 2.31, –, 0.80). Leg I: 15.32 (4.38, 2.12, 3.48, 3.25, 2.09). Leg II: 13.73 (3.91, 1.86, 3.19, 2.77, 2.00). Leg III: 13.11 (3.47, 1.85, 2.40, 3.31, 2.08). Leg IV: 16.63 (4.40, 1.95, 3.39, 4.60, 2.29). Leg I: tibia incrassate, metatarsus slightly curved retroventrally (Fig. 28).

Spination. Palp: femur d0(1)–1–1–1, pd0–1–1; patella p1–1; tibia d1–1–1, r0–1(0)–1, pv1–1–1, rv1–1–1; cymbium d2(4). Leg I: femur d1–1–1–1, pd0–1–1; rd 0–0–1; tibia p1–1–0, pv1–1; rv1–1–M–M; metatarsus v0–0–2. Leg II: femur d1–1–1–1; pd1–1–1; patella p1–1; tibia p1–1–1, v1–2–1(2)–3; metatarsus p0–1–0; v1–2–2. Leg III: femur d1–1–1–1, pd1–1–1, rd1–1–1; patella p1–1, r1; tibia d1–1–1, p1–1–1, r1–1–1, v2–2–3; metatarsus d1–1–1, p1–1–1, r1–1–1, v2–2–3. Leg IV: femur d1–1–1–1, pd1–1–1, rd1–1–1; patella p1, r1; tibia d1–1–1(0), p1–1–1, r1–1–1, v2–2–3; metatarsus pd1–1–1, p1–1–1, r1–1–1, v2–2–2–3. Patella I and tarsi I–IV aspinose.

Scopula: entire distal 2/3 and 1/2 on metatarsi I and II, respectively; entire on tarsi I and II, widely divided by setae on tarsus III; vestigial on tarsus IV. Paired claws on tarsi I-III and IV with 8–10 and 8–10 teeth per row, respectively. Trichobothria: 2 rows of 7–8 per row on tibiae, 8–11 on metatarsi, 8–9 on tarsi, 6 on cymbium.

Palpal tibia long, cymbium with few short spines (Fig. 34). Bulb without ridges; embolus long, acuminate and slightly twisted (Fig. 42).

Spinnerets. PMS: length 0.41; diameter 0.14. PLS: maximum diameter 0.46; length of basal, medial and apical segments 0.70, 0.44, 0.42; total length 1.56; apical segment short-digitiform.

Female unknown.

#### Variability.

Carapace length in males varies from 4.80 to 5.43 (n=12).

#### Distribution.

CHINA: Yunnan Province (Fig. 1).

**Figures 14–17.**
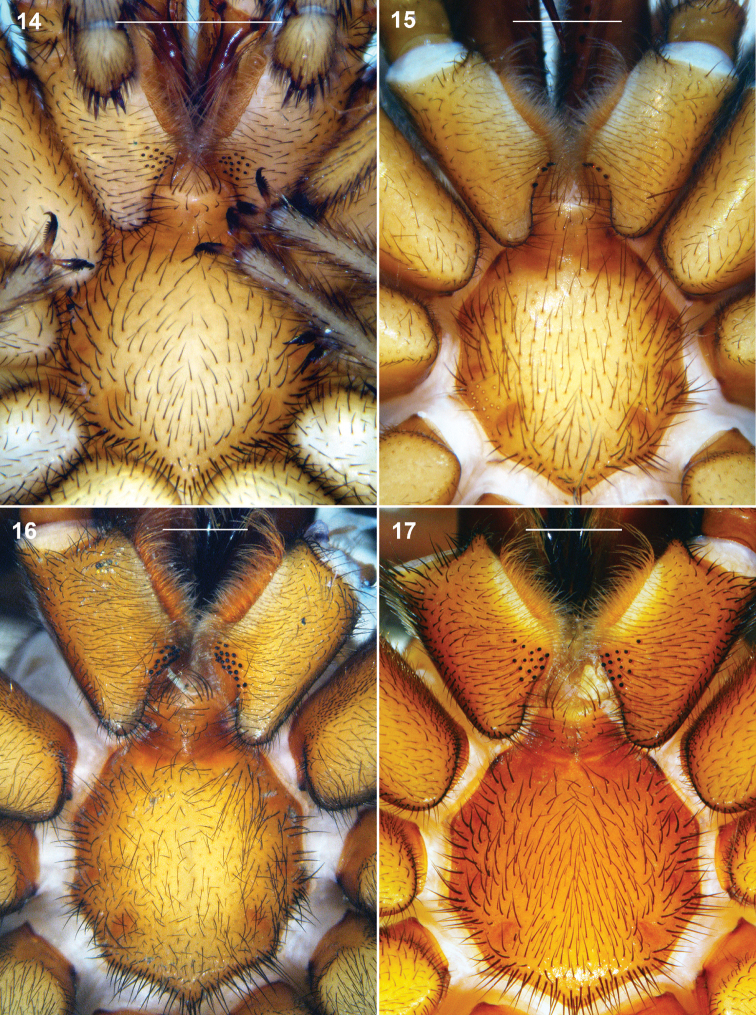
*Sinopesa* and *Raveniola*, holotype (**15, 17**) and conspecific (**14, 16**) males: sternum, labium and maxillae, ventral view **14** *Sinopesa maculata* **15** *Raveniola guangxi* **16** *Raveniola hebeinica* **17** *Raveniola montana* sp. n. (scale bar = 1 mm).

**Figures 18–21.**
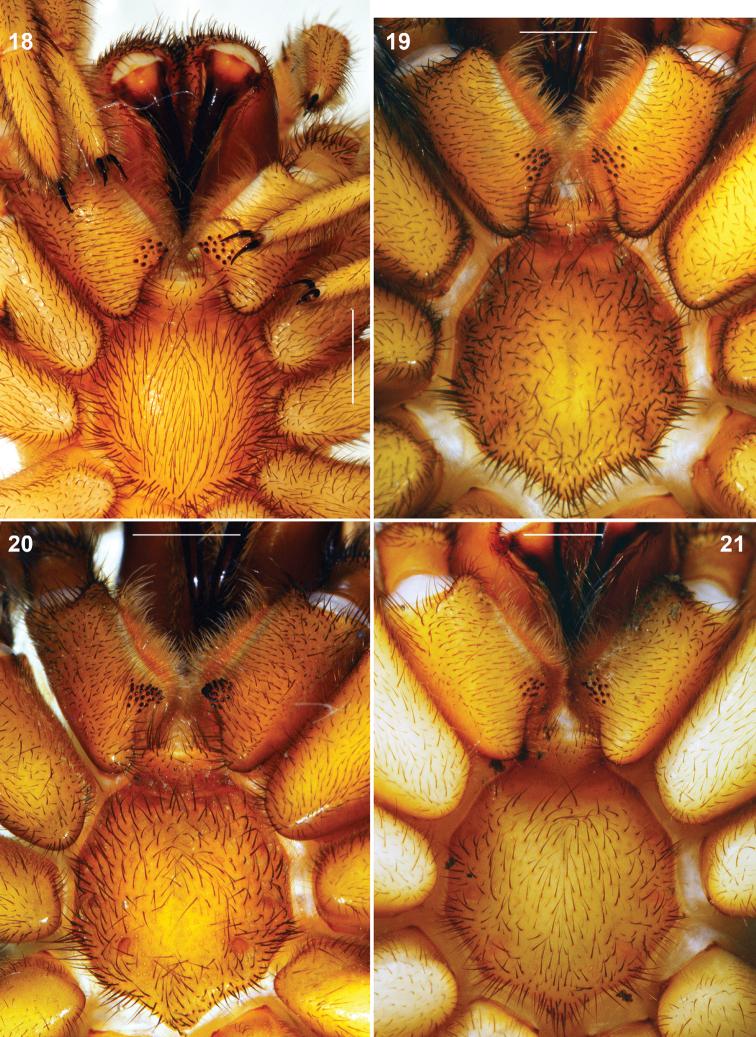
*Raveniola*, holotype (**21**) and paratype (**18–20**) males (**19–21**) and female (**18**): sternum, labium and maxillae, ventral view **18** *Raveniola montana* sp. n. **19** *Raveniola shangrila* sp. n. **20** *Raveniola songi*sp. n. **21*** R. yunnanensis* sp. n. (scale bar = 1 mm).

**Figures 22–25.**
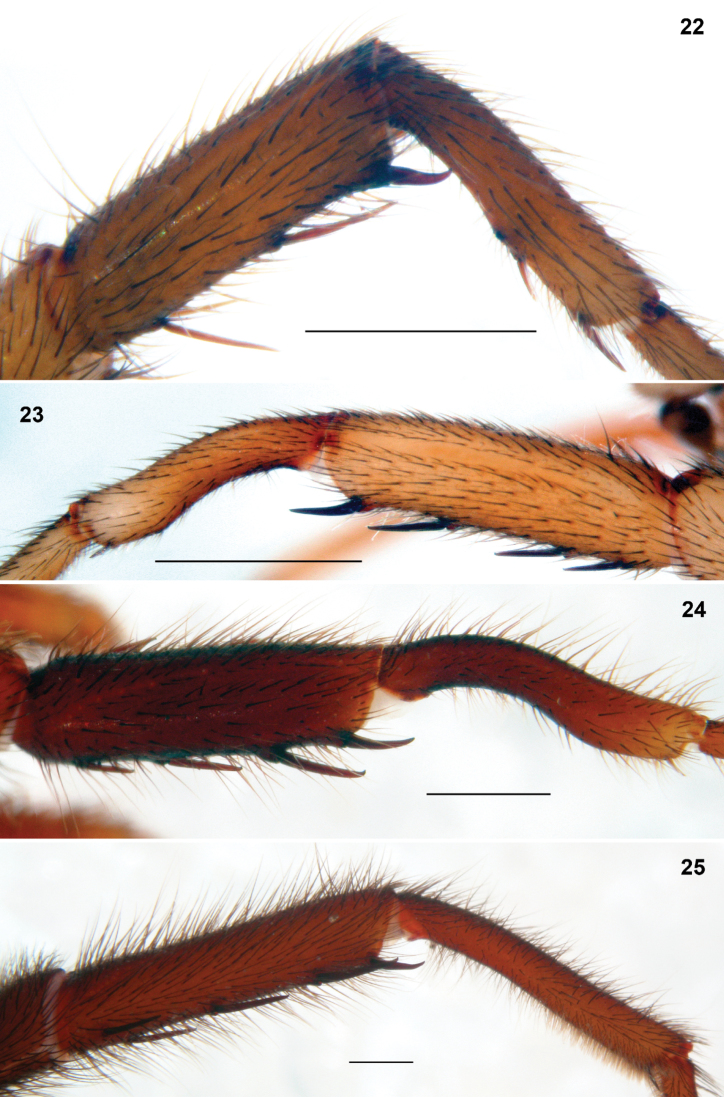
*Entypesa*, *Sinopesa* and *Raveniola*, holotype (**24**) and conspecific (**22, 23, 25**) males: tibia and metatarsus I, retrolateral view. **22** *Entypesa schoetedeni* **23** *Sinopesa maculata* **24** *Raveniola guangxi* **25** *Raveniola hebeinica* (scale bar = 1 mm).

### Raveniola yunnanensis sp. n.

urn:lsid:zoobank.org:act:BCC3B9B7-A813-46D3-9DB1-3C9884038FCA

http://species-id.net/wiki/Raveniola_yunnanensis

Figs 13 21 29 35 43

#### Types.

Holotype ♂ – CHINA: Yunnan Province, Finchuiyanou Mts. 40 km NNW of Baoshan, 25°28'54"N, 99°05'05"E, 3200 m, 10.05.2005, coll. I. Kabak & I. Belousov (IZAS).

#### Etymology.

The specific epithet is given after the name of the inhabited region (Yunnan).

#### Diagnosis.

Males differ from all other Chinese congeners by lighter body colouration and longer legs (tibia I 5.5 times as longer than wide vs. 4–5 times in other species) and more spinose embolus armed with ca. 25–30 spines (vs. 3–7).

#### Description.

Male (holotype). Body length 12.85. Colour in alcohol: carapace, chelicerae, palps and first pair of legs dorsally intense yellowish orange; eye tubercle with darker spots surrounding AMEs and lateral eyes; sternum, labium, maxillae and legs light yellowish orange; abdomen dorsally uniformly light grey, ventral abdominal surface and spinnerets pale greyish yellow.

Carapace (Fig. 13) 5.73 long, 4.60 wide; covered with moderately dense and thin semi-adpressed dark hairs. Eye diameters (AME, ALE, PLE, PME): 0.17 (0.24), 0.23, 0.13, 0.08/0.09. Interdistances: AME–AME 0.15 (0.10), ALE–AME 0.13 (0.10), ALE–PLE 0.12, PLE–PME 0.05, PME–PME 0.57. Cheliceral furrow with 9–10 promarginal teeth and 6–7 mesobasal denticles. Labium (Fig. 21) 0.43 long, 0.78 wide. Maxillae with 13–16 cuspules in compact area confined to basal maxillary edge. Sternum 2.32 long, 2.34 wide. Palp: 8.29 (3.52, 1.54, 2.41, –, 0.82). Leg I: 18.72 (5.17, 2.86, 4.49, 3.77, 2.43). Leg II: 17.70 (4.84, 2.52, 4.24, 3.67, 2.43). Leg III: 16.52 (4.24, 2.05, 3.52, 4.10, 2.61). Leg IV: 21.57 (5.22, 2.30, 5.02, 6.26, 2.77). Leg I: tibia 5.48 times longer than broad, slightly arcuate, metatarsus slightly curved retroventrally (Fig. 29).

Spination. Palp: femur d1–1–1–1, pd1–1–1; patella p1; tibia d2–1–2, p1–0–1–1–1, pv1–1–2–1, rv1–1–0; cymbium d *ca*.20. Leg I: femur d1–1–1(0)–0, pd1–1–1; rd 1(0)–1–1; patella p1; tibia p1–1–0, pv1–0–1–1, rv 1–1–0–M–M; metatarsus v0–1–2. Leg II: femur d1–1–1(0)–0; pd1–1–1; tibia p1–1–1, v2(3)–2–3; metatarsus p1–1; v2–2–3. Leg III: femur d1–1–1–1, pd1–1–1, rd1–1–1; patella p1, r1; tibia d1–1–1, p1–1–1, r1–1–1–1, v2–2–3; metatarsus d1–1–1, p1–1–2, r1–1–1–1, v2–2–3. Leg IV: femur d1–1–1–1, pd1–1–1, rd1–1–1; patella p1; tibia d1–1–0, p1–1–1, r1–1–1–1, v2–2–3; metatarsus pd1–1–1, p1–1–1, r1–1–1–1, v2–1–1–3. Tarsi I–IV aspinose.

Scopula: moderately dense and long – entire covering whole ventral metatarsus I and distal 2/3 of metatarsus II, entire on tarsi I–II, divided by setae on tarsus III; widely divided and vestigial on metatarsus III and tarsus IV. Paired claws: legs I–III with 6–8 teeth, leg IV with 9 teeth in two rows on each claw. Trichobothria: 2 rows of 6–8 per row on tibiae, 10–12 on metatarsi, 8–10 on tarsi, 6 on cymbium.

Palpal tibia long; cymbium strongly spinose (Fig. 35). Bulb without ridges; embolus gradually tapering and slightly twisted (Fig. 43).

Spinnerets. PMS: length 0.30; diameter 0.12. PLS: maximum diameter 0.48; length of basal, medial and apical segments 0.83, 0.62, 0.77; total length 2.22; apical segment digitiform.

Female unknown.

#### Distribution.

CHINA: Yunnan Province (Fig. 1).

**Figures 26–29.**
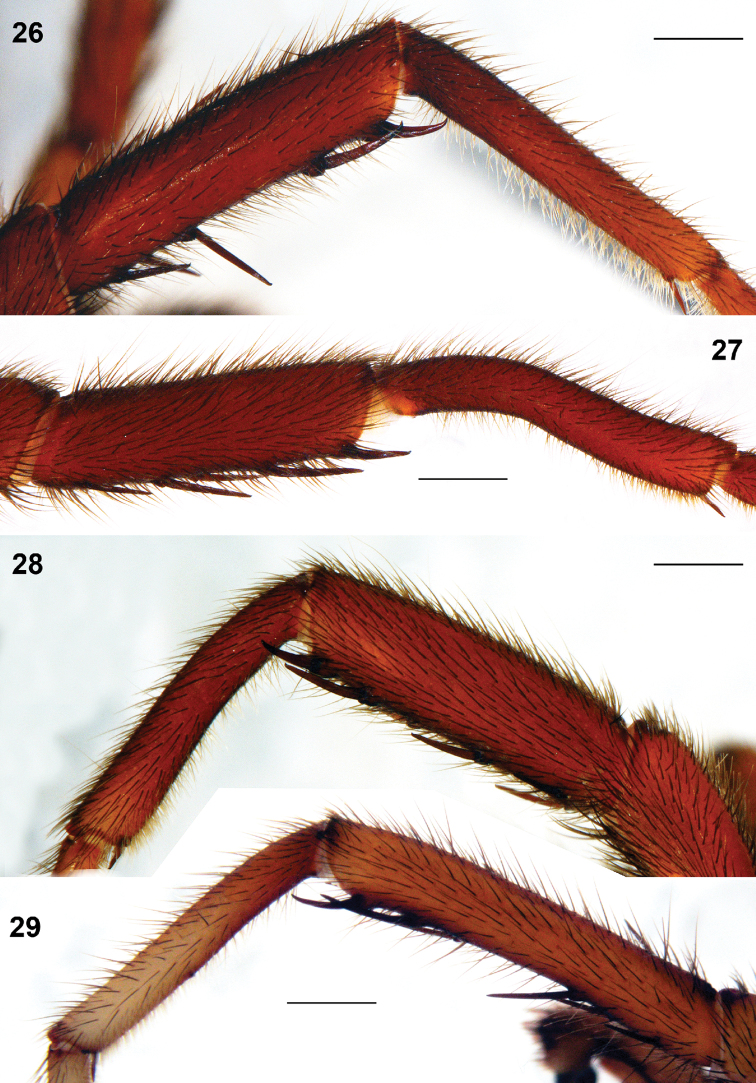
*Raveniola*, holotype (**26, 29**) and paratype (**27, 28**) males: tibia and metatarsus I, retrolateral view. **26** *Raveniola montana* sp. n. **27** *Raveniola shangrila* sp. n. **28** *Raveniola songi*sp. n. **29** *Raveniola yunnanensis* sp. n. (scale bar = 1 mm).

**Figures 30–35.**
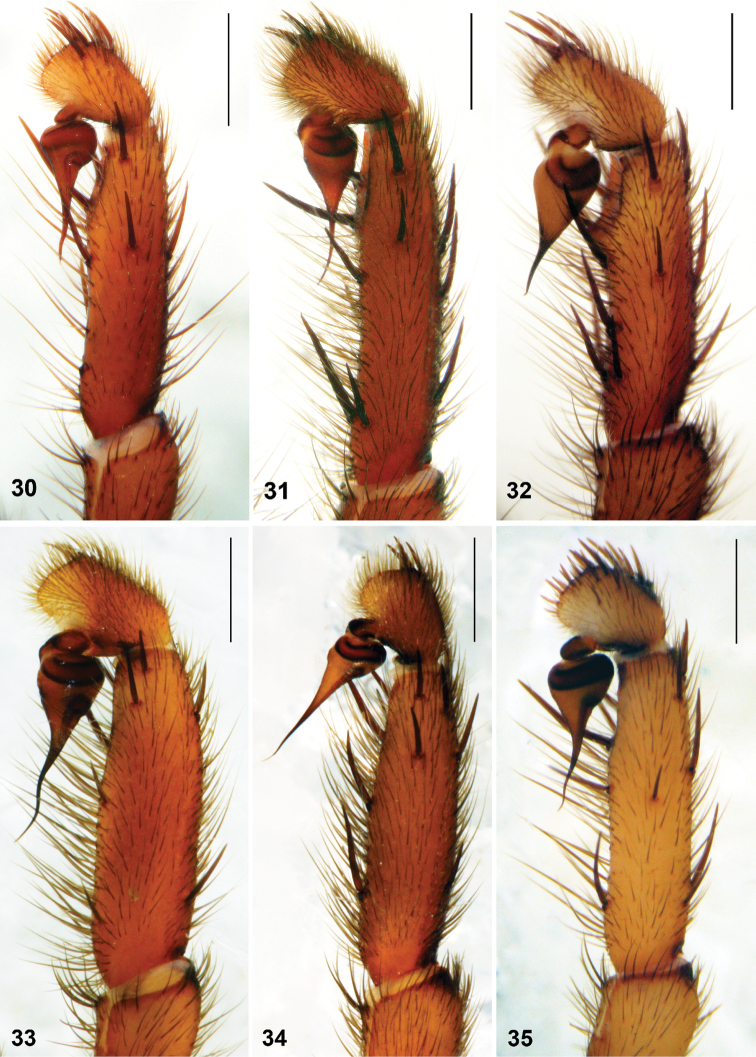
*Raveniola*, holotype (**30, 32, 35**), paratype (**33, 34**) and conspecific (**31**) males: palpal tibia, cymbium and bulbus, retrolateral view. **30** *Raveniola guangxi* **31** *Raveniola hebeinica* **32** *Raveniola montana* sp. n. **33** *Raveniola shangrila* sp. n. **34** *Raveniola songi*sp. n. **35** *Raveniola yunnanensis* sp. n. (scale bar = 1 mm).

**Figures 36–39.**
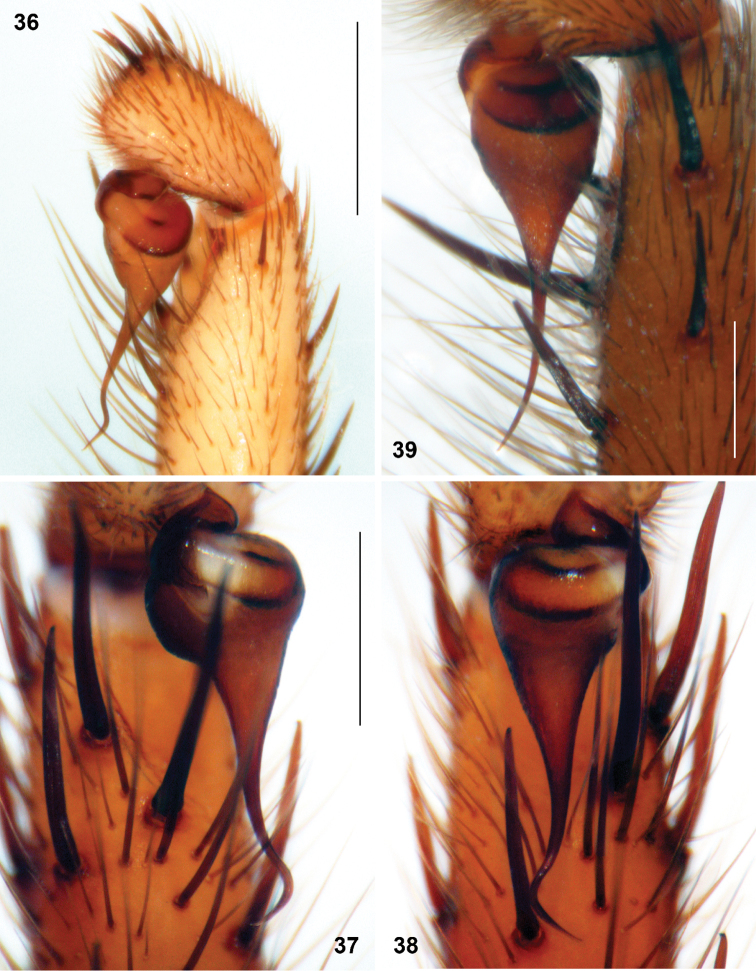
*Sinopesa* and *Raveniola*, holotype (**37, 38**) and conspecific (**36, 39**) males: bulbus, ventral (**38**) retroventral (**36, 37**) and retrolateral (**39**) view. **36** *Sinopesa maculata* **37, 38** *Raveniola guangxi* **39** *Raveniola hebeinica* (scale bar = 0.5 mm). Note: Figs **37** and **38** show right and left palpi of the same specimen, respectively.

**Figures 40–43.**
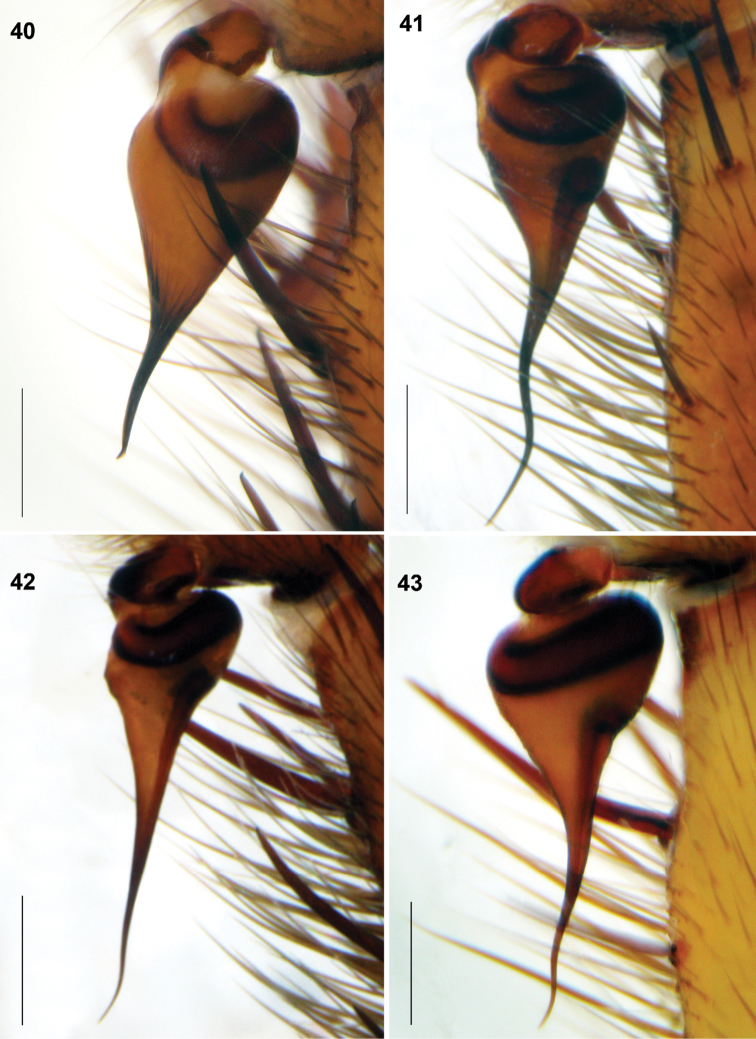
*Raveniola*, holotype (**40, 43**) and paratype (**41, 42**) males: palpal bulbus, retrolateral view. **40** *Raveniola montana* sp. n. **41** *Raveniola shangrila* sp. n. **42** *Raveniola songi*sp. n. **43** *Raveniola yunnanensis* sp. n. (scale bar = 0.5 mm).

### Raveniola xizangensis

(Hu & Li, 1987)

http://species-id.net/wiki/Raveniola_xizangensis

Figs 49, 50

Brachythele xizangensis Hu & Li, 1987: 315–318, 385, figs 1.1–7, 2.1–2 (♂♀); Hu 2001: 65-67, figs 7.1–4 (♂♀).
Raveniola xizangensis : Song et al. 1999: 40, 47, figs 17I–J (♂♀); Zonstein 2000: 50.

#### Types.

♀ holotype and ♂ paratype from Jancha County, Tibet; dep. BDSU, not examined.

#### Diagnosis.

*Raveniola xizangensis* differs from all other Chinese congeners as well as from north-west-Himalayan *Raveniola concolor* Zonstein, 2000 by the developed subapical embolic keel in males and the bifurcate basal receptacle in females (cf. Figs 37–43, 47-50, and Zonstein 2000, figs 4–6, respectively).

#### Description.

This largest Chinese nemesiid with carapace 11-12 mm long was well described by Hu and Li (1987). Bulbus and spermathecae as shown in Figs 49 and 50.

#### Distribution.

Known only from the type locality – Jancha County, Tibet (see Fig. 1, *Raveniola* loc. 1).

**Figures 44–46.**
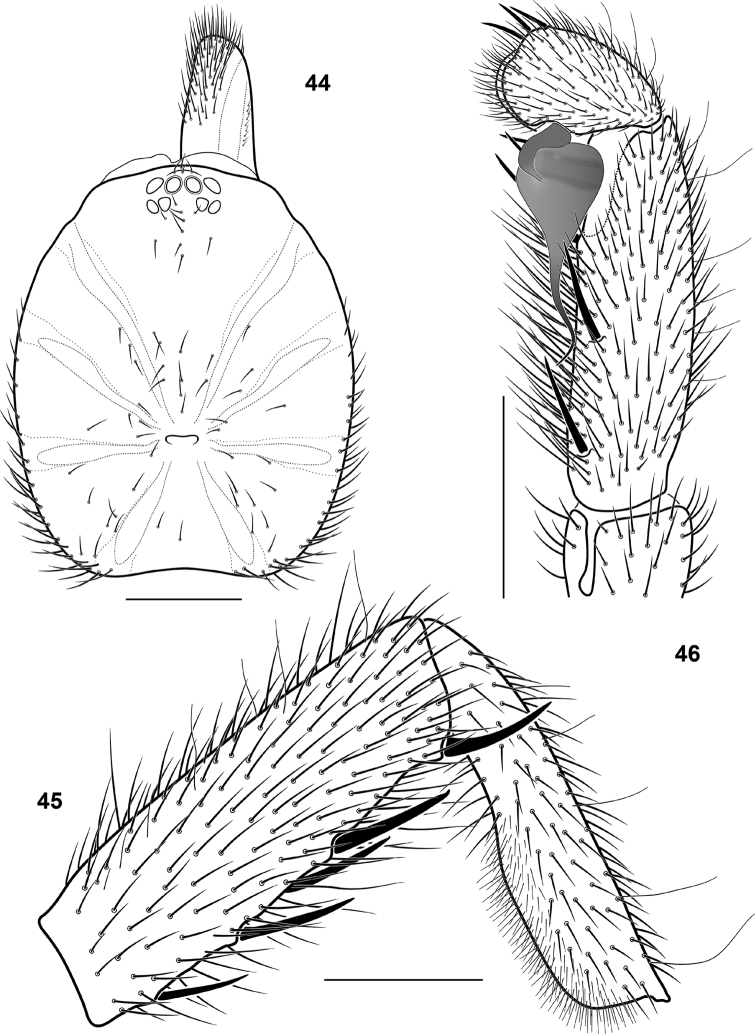
*Sinopesa chinensis* (Kulczyński, 1901) comb.n., conspecific male (sensu Kritscher 1957): structures, dorsal (**44**) and retrolateral (**45, 46**) view. **44** carapace **45** tibia and metatarsus I **46** palpal tibia, cymbium and bulbus (scale bar = 1 mm).

**Figures 47–50.**
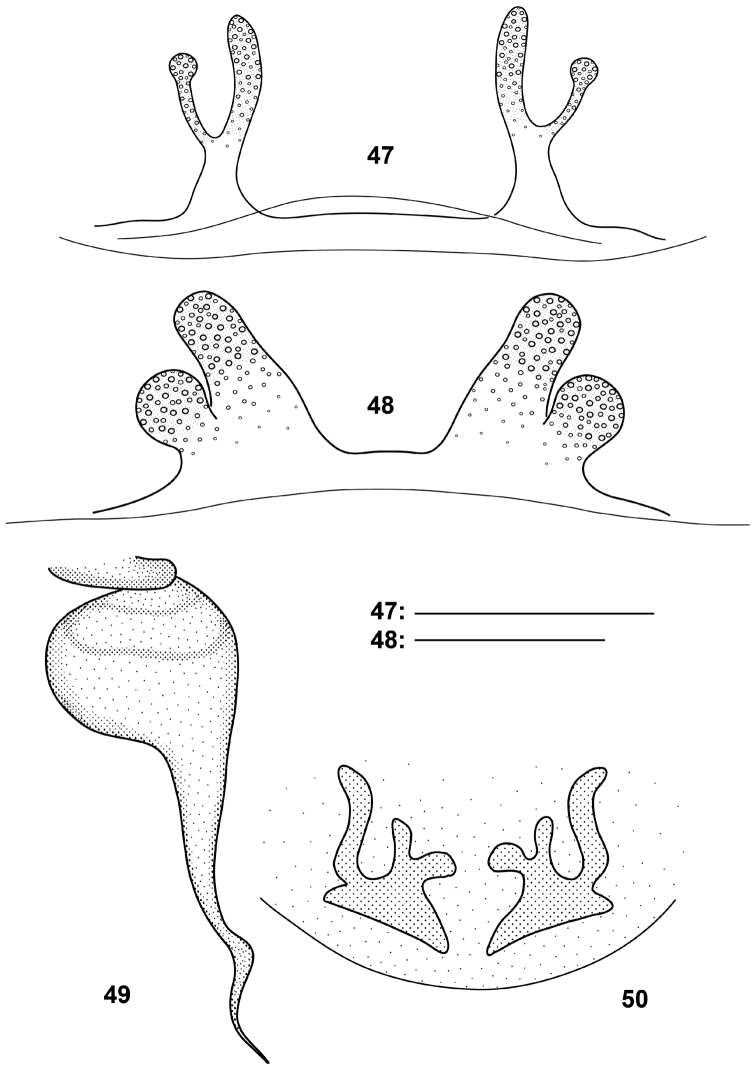
*Raveniola*, bulbus (**49,** holotype male, ventral view) and spermathecae (**47, 48, 50,** conspecific/paratype females, ventral view): **47** *Raveniola hebeinica* **48** *montana* sp. n. **49, 50** *Raveniola xizangensis* (from Hu and Li (1987), modified); scale bar = 0.25 mm.

## Discussion

Three nemesiid genera have been reported from China to date. The first is *Nemesia* Audouin, 1826, represented here by the enigmatic *Nemesia sinensis*, known only from the holotype female (Pocock 1901). Judging from the description, this species possesses, unlike most nemesiids, spinose leg tarsi and sparcely spinose leg IV and thus might actually belong to the Cyrtaucheniidae. In addition, all correctly described *Nemesia* are known westwardly the Caucasus. The second genus is *Raveniola*, which, according to the Chinese authors (Song et al. 1999; Xu and Yin 2002), includes the majority of the regional nemesiids sharing the diagnostic characters indicated by Zonstein (1987): the sequential retroventral megaspines in males and divided spermathecae in females. Finally, at least one Chinese nemesiid was referred to the endemic East-Asian genus *Sinopesa* (see Raven and Schwendinger 1995).

While establishing *Sinopesa*, Raven and Schwendinger separated it from the related genus *Entypesa* on the basis of two characters: the absence of PMS and of serrula. The apical segment of PLS in *Sinopesa* spp. was found to be digitiform. Later, Shimojana and Haupt (2000) described *Sinopesa kumensis* from Ryuku islands, Japan, as definitely related to the type species *Sinopesa maculata* Raven & Schwendinger, 1995, but possessing PMS though in the reduced form. One more unequivocal character of *Sinopesa* that has not been specially noted by the above-mentioned authors is a retroventral position of male megaspines; also shared by some other nemesiids: by the African genera *Entypesa* (Figs 3, 22), *Lepthercus* and *Pionothele* (whereas in the definitely related *Hermacha* the male tibia I appears to be unmodified) and, as just stated, by *Raveniola*. At least for the latter genus, the very construction was shown to be connected with the specific way of the female fixation during the mating (Zonstein 2002).

It should be noted that in the mentioned African genera the spinneret morphology retains the more plesiomorphic condition: PMS are relatively large and fully functional, and the apical segment of PLS in all these genera except *Pionothele* is long and slender (digitiform). The spinneret morphology in *Sinopesa* is noted above. In *Raveniola* species PMS are small to tiny; within the species described to date they are absent in *Raveniola fedotovi* (Charitonov, 1946) and *Raveniola kopetdaghensis* (Fet, 1984); the apical segment of PLS varies, with few exceptions (see Zonstein 2009), from short-digitiform to triangular.

Although *Sinopesa* and *Raveniola* have never hitherto been compared to one another, they share a number of apomorphies that might bring them closer to each other than to the mentioned genera. Some of these characters, such as the absence of the maxillary serrula and the metatarsal preening combs, are also shared with *Lepthercus* and *Pionothele*; whereas other features appear to be unique. Males of both Asian nemesiid genera share the presence of 2–3 (vs. one in the mentioned African nemesiids, as shown in Fig. 22) retroventral megaspines on tibia I. The congeneric females possess the divided spermathecae (that are entire in *Entypesa* and *Hermacha* - see Raven 1985; for *Lepthercus* and *Pionothele* known from males this character is uncertain). The most important shared feature is that within the Mygalomorphae only males of *Raveniola* and *Sinopesa* are found to possess the intercheliceral tumescence located ventrally (not prolaterally) and confined to the cheliceral furrow.

Differences between Chinese members of *Sinopesa* and *Raveniola* are summarised below:

In the course of this study the characters of three Chinese nemesiids previously placed in *Raveniola* were found to correspond to the diagnostic features of *Sinopesa*. Hence, they are transferred here to the latter genus: *Sinopesa chinensis* (Kulczyński, 1901) comb. n., *Sinopesa sinensis* (Zhu & Mao, 1983) comb. n. and *Sinopesa chengbuensis* (Xu & Yun, 2002) comb. n. Moreover, using the same definitive criteria, one of the existing members of *Sinopesa* should be transferred to *Raveniola*: *Raveniola guangxi* (Raven & Schwendinger, 1995) comb. n. The current generic position of *Raveniola xizangensis* (Hu & Li, 1987) and *Raveniola hebeinica* Zhu et al., 1999, whose features do not contradict the generic characters, is presently confirmed.

Being compared by shape of the male embolus and female spermathecae with other *Raveniola* species, the Chinese representatives showed a closer similarity to the Central Asian congeners and especially to North-West Himalayan *Raveniola concolor* (cf. Zonstein 2000, figs 4–6), as well as it was possible to expect.

## Prospective

Seven true Chinese species of *Raveniola* were revealed in course of this study, which engages here, however, only with the material and information previously available. Currently, we cannot estimate the true diversity of *Raveniola* species within the country, but expect it to be much higher. This expectation is based, first and foremost, on the fact that many parts of the region were not specially investigated; while a rather small amount of material from two geographically close localities has already revealed four new species. An additional possible factor relates to the limited and often sympatric character of their distribution, specific to this group of spiders. Numerous mountain ridges in central and southern parts of China (which provide both the mosaic character and richness of habitats) and lack of special collection efforts suitable for these mygalomorphs, reinforces the prediction regarding their probably higher species diversity.

The prospective areas in which new findings might be expected are the provinces lying between the two main groups of the known localities (see Fig. 1), especially Sichuan and southern part of Gansu. It should be noted that the members of this genus may also occur in the furthest north-western part of China. According to our data (Zonstein, in prep.), some *Raveniola* species were observed in Kyrgyzstan, inhabiting Alai and Trans-Alai Mt. Ridges, both adjoining Xingjian.

## Supplementary Material

XML Treatment for Raveniola

XML Treatment for Raveniola guangxi

XML Treatment for Raveniola hebeinica

XML Treatment for Raveniola montana

XML Treatment for Raveniola shangrila

XML Treatment for Raveniola songi

XML Treatment for Raveniola yunnanensis

XML Treatment for Raveniola xizangensis
